# Identification of Tomato Proteins That Interact With Replication Initiator Protein (Rep) of the Geminivirus TYLCV

**DOI:** 10.3389/fpls.2020.01069

**Published:** 2020-07-15

**Authors:** Francesca Maio, Tieme A. Helderman, Manuel Arroyo-Mateos, Miguel van der Wolf, Sjef Boeren, Marcel Prins, Harrold A. van den Burg

**Affiliations:** ^1^ Molecular Plant Pathology, Swammerdam Institute for Life Sciences (SILS), University of Amsterdam, Amsterdam, Netherlands; ^2^ Laboratory of Biochemistry, Wageningen University, Wageningen, Netherlands; ^3^ Keygene N.V., Wageningen, Netherlands

**Keywords:** geminivirus, *Tomato yellow leaf curl virus*, viral replication, proteomics, PCNA, Rep, interactome

## Abstract

Geminiviruses are plant-infecting DNA viruses that reshape the intracellular environment of their host in order to create favorable conditions for viral replication and propagation. Viral manipulation is largely mediated *via* interactions between viral and host proteins. Identification of this protein network helps us to understand how these viruses manipulate their host and therefore provides us potentially with novel leads for resistance against this class of pathogens, as genetic variation in the corresponding plant genes could subvert viral manipulation. Different studies have already yielded a list of host proteins that interact with one of the geminiviral proteins. Here, we use affinity purification followed by mass spectrometry (AP-MS) to further expand this list of interacting proteins, focusing on an important host (tomato) and the Replication initiator protein (Rep, AL1, C1) from *Tomato yellow leaf curl virus* (TYLCV). Rep is the only geminiviral protein proven to be essential for geminiviral replication and it forms an integral part of viral replisomes, a protein complex that consists of plant and viral proteins that allows for viral DNA replication. Using AP-MS, fifty-four ‘high confidence’ tomato proteins were identified that specifically co-purified with Rep. For two of them, an unknown EWS-like RNA-binding protein (called Geminivirus Rep interacting EWS-like protein 1 or GRIEP1) and an isoform of the THO complex subunit 4A (ALY1), we were able to confirm this interaction with Rep *in planta* using a second method, bimolecular fluorescence complementation (BiFC). The THO subunit 4 is part of the THO/TREX (TRanscription-EXport) complex, which controls RNA splicing and nuclear export of mRNA to the cytoplasm and is also connected to plant disease resistance. This work represents the first step towards characterization of novel host factors with a putative role in the life cycle of TYLCV and possibly other geminiviruses.

## Introduction

During the past twenty years, geminiviruses (family *Geminiviridae*) have emerged as one of the most destructive plant-infecting virus families (Rojas et al., 2005). They infect numerous plant species causing significant yield losses in many economically important crops, both monocots and dicots (Mansoor et al., 2003; Mansoor et al., 2006). Geminiviruses are transmitted between host plants by insect vectors, for example leafhoppers, treehoppers and whiteflies. Based on these vectors and their genome organization, they are currently classified into nine genera (Zerbini et al., 2017). Viral transmission of the largest genus, *Begomoviruses* (>380 species), is mediated by the whitefly *Bemisia tabaci*, a highly polyphagous and worldwide spread insect (Zhao et al., 2019). Correspondingly, *Begomoviruses* have a wide host range, infecting many plant species found in the (sub)tropical regions and in more temperate climate zones (Navas-Castillo et al., 2011). Traditionally, begomovirus infestation was (to some extent effectively) controlled in the field by a combination of (a) insecticide rotation to control whitefly populations and (b) agricultural practices that reduce virus reservoirs in weeds. The success rate of these combined measures varies a lot between crops, cropping systems, and geographical regions (Rojas et al., 2018). To support these aforementioned control measures, they need to be complemented with genetic resistance in crops. So far, few resistance traits against the *Tomato yellow leaf curl virus* (TYLCV) have been identified and introduced in cultivated crops. Among them were the allelic genes *Ty-1* and *Ty-3*, which encode an RNA-dependent RNA polymerase (Verlaan et al., 2013). Introgression of these genes into tomato cultivars conferred resistance to TYLCV by increasing cytosine DNA methylation of the viral genome, thus acting through viral transcriptional gene silencing (Caro et al., 2015). Another example of a TYLCV resistance locus is *Ty-2*, which was genetically linked to the gene *TYNBS1* that encodes a NB-LRR (Nucleotide-Binding domain and Leucine-Rich Repeat-containing) type plant immune receptor (Yamaguchi et al., 2018). However, following the introduction of the resistance genes *Ty-1/3* and *Ty2* in tomato cultivars, resistance-breaking strains of TYLCV have emerged in recent years under field conditions, suggesting that these genes do not confer durable resistance against TYLCV (Verlaan et al., 2013; Butterbach et al., 2014; Belabess et al., 2016; Ohnishi et al., 2016). To increase our arsenal of genetic resources for breeding and to help reduce insecticide usage, new genetic strategies are thus needed. One strategy is to screen for resistance based on natural variation in *Susceptibility* (S) genes (van Schie and Takken, 2014), in this particular case for host genes that are critical for geminiviruses to complete their life cycle in plant cells.

Geminiviruses are typified by their capsid composed of a twinned icosahedral particle that encapsidates the viral genome composed of circular single-stranded DNA (ssDNA) molecule. Within the genus *Begomovirus*, this genome can either be *monopartite* (~2.8 kb) encoding six proteins that jointly control viral replication, movement, transmission, and pathogenesis, or *bipartite* with an **‘**A *component* **’** that encodes five or six viral proteins and a **‘**B *component* **’** that encodes for two additional proteins (both components being 2.5–2.8 kb in size) (Jeske, 2009). Geminivirus infections start foremost in terminally differentiated cells, i.e. plant cells that are in the G1 phase of the mitotic cell cycle or in the G phase of the endocycle—a variation of the cell cycle characterized by increased ploidy levels and cell expansion without cell division. In order to orchestrate replication of its viral genome, the virus thus needs to reprogram the host cell cycle to promote progression into the S phase of the cell cycle (Hanley-Bowdoin et al., 2004; Hanley-Bowdoin et al., 2013). Only one viral protein, the Replication initiator protein (Rep), is strictly required for viral replication to occur. In fact, Rep is the most conserved geminiviral protein and it is critical for assembly of *viral replisomes*, a complex of viral and host proteins that forms a DNA replication fork at viral DNA (Rizvi et al., 2015; Ruhel and Chakraborty, 2019). Numerous studies have been attempted to characterize the composition and mode-of-action of this viral replisome. At least one other viral protein, REn (Replication enhancer protein), is known to promote viral replication and as such it is likely also part of viral replisomes (Sunter et al., 1990).

Previous studies support that both Rep and REn interact with host factors in order to create a cellular environment favorable for virus replication, e.g. by up-regulating expression of several host DNA polymerases and DNA replication accessory proteins (Gutierrez, 2000a; Gutierrez, 2000b). One critical component of viral replisomes is the protein PCNA (Proliferating cell nuclear antigen), which acts as a processivity factor of eukaryotic DNA polymerases and forms a DNA clamp at replication forks (Castillo et al., 2003; Morilla et al., 2006). PCNA is a highly conserved protein in eukaryotes that controls the cell cycle, DNA replication and DNA repair pathways (Choe and Moldovan, 2017). Different studies have shown that both Rep and REn from different geminiviruses (TYLCV, *Tomato yellow leaf curl Sardinia virus* [TYLCSV], *Tomato Golden Mosaic Virus* [TGMV], *Indian mung bean yellow mosaic virus* [IMYMV]) interact with PCNA (Castillo et al., 2003; Bagewadi et al., 2004; Settlage et al., 2005) and that Rep from two different *Begomoviruses* (TYLCV and TGMV) manipulates the SUMOylation status of PCNA—a conserved protein modification of PCNA that directly modulates PCNA function (Arroyo-Mateos et al., 2018). Other host factors that are recruited by members of the Rep protein family to viral replisomes include among others the Replication factor C, RFC (Luque et al., 2002), Replication protein A32, RPA32 (Singh et al., 2007), the recombination and DNA repair related proteins Rad51 and Rad54 (Kaliappan et al., 2012; Suyal et al., 2013a) and the Minichromosome maintenance protein 2, MCM2 (Suyal et al., 2013b). Moreover, Rep also reprograms the host cell cycle by binding transcriptional regulators of the cell cycle like the Retinoblastoma-related protein (RBR) (Xie et al., 1996; Kong et al., 2000) and several members of the NAC family of transcription factors (GRAB1, GRAB2, NAC083) (Suyal et al., 2014) and by interacting with proteins involved in post-translational modification mechanisms, such as ubiquitination and sumoylation (Castillo et al., 2004; Sánchez-Durán et al., 2011; Kushwaha et al., 2017). Besides, Rep from TGMV was also shown to suppress expression of certain viral genes by binding to the viral DNA (Eagle et al., 1994; Shung and Sunter, 2007). Finally, Rep from different geminiviruses was shown to suppress methylation‐mediated transcriptional gene silencing (TGS) by reducing the expression of the responsible DNA methyltransferases (MET1 and CMT3) in the plant species *Nicotiana benthamiana* and *Arabidopsis thaliana* (Rodríguez-Negrete et al., 2013).

Clearly, the geminivirus-plant interaction is intrinsically complex involving a multitude of cellular pathways (Hanley-Bowdoin et al., 2013). To better understand how geminiviruses redirect these pathways, we need to characterize in more detail the protein network of the viral proteins with host proteins. A detailed characterization of this network will aid in the identification of key steps needed for viral replication and allows exploitation of this knowledge to attain durable and broad resistance to these viruses e.g. *via* mutagenesis or natural variation of the underlying genes to ultimately disrupt the viral life cycle. Affinity-based purification followed by mass spectrometry-based protein identification (AP-MS) forms a well-suited technique to characterize the composition of these protein complexes under native conditions (Gavin et al., 2011; Dunham et al., 2012). In the context of plant–virus interactions, AP-MS has been used for example to define the interactome of the NIa protein from the potyvirus *Tobacco etch virus* (Martínez et al., 2016) and to characterize the global landscape of interactions between TYLCV and one of its host plants, *N. benthamiana* (Wang et al., 2017a).

Here we expand this list of potential host-interacting proteins by using an AP-MS approach to compose a list of **‘**high-confidence**’** tomato proteins that co-purify with TYLCV Rep in combination with tomato PCNA. For a small subset of these tomato proteins, (i) an EWS (Ewing Sarcoma protein)-like RNA-binding protein (hereafter named Geminivirus Rep-interacting EWS-like protein 1 [GRIEP1]), and (ii) the THO/TREX (TRanscription-EXport) subunit 4A (ALY1), we confirmed that they interact with Rep and PCNA *in planta* using an independent method, *i.e.* the bimolecular fluorescence complementation (BiFC). The THO/TREX complex is involved in mRNA splicing and subsequent export of mRNAs to the cytoplasm and several lines of evidence indicate that nucleocytosolic mRNA transport contributes to the regulation of plant immunity (Ehrnsberger et al., 2019). Moreover, ALY proteins have been shown to play an important role during viral infections (Xu et al., 2020). This work thus extends our inventory of Rep interactions and represents a first step towards characterization of these host factors.

## Materials and Methods

### Tomato Protoplast Isolation and Transfection

Protoplasts were isolated from *in vitro* shoot cultures of tomato as described (Shahin, 1985; Tan et al., 1987). A total of 4 × 10^7^ protoplasts per sample were subjected to Polyethylene-glycol (PEG4000) mediated transformation (Negrutiu et al., 1987) with the following plasmids: (i) pK7FWG2 (Karimi et al., 2002) containing *Rep* from TYLCV (Genbank ID: FJ956702.1) fused to enhanced *GFP* (EGFP) (referred to as Rep-GFP), (ii) Rep-GFP + pJL-TRBO (Lindbo, 2007) containing tomato (*Sl*)*PCNA* fused to the FLAG tag at its N-terminus, referred to as FLAG-PCNA (positive control for interaction), and (iii) FLAG-PCNA as a negative control. Transfected protoplasts were incubated in Gamborg B5 (Duchefa Biochemie) liquid medium at 25°C overnight in dark conditions. Three independent protoplasts isolations and DNA transfections were performed on three different days. The next day the protoplasts were collected in 1.5 ml reaction vials by centrifugation (5 min at 85 g) and frozen at −80°C till further usage.

### Protoplasts Protein Extraction and Immunoprecipitation

Protoplasts were defrosted and resuspended in 1 ml per 10^7^ protoplasts of Triton X-buffer (20 mM Tris–HCl pH 7.5, 10 mM KCl, 10% glycerol, 1 mM DTT, 10 mM MgCl_2_, 2 mM EDTA, 1% Triton X-100, 1 mM PMSF, and 1 mM NaF). Protoplast mixtures were incubated on ice for 30 min and sonicated twice for 15 s. NaCl 2M solution was added to the protoplast suspension at a final concentration of 420 mM and tubes were incubated on ice for 1 h with occasional mixing. All protein sample extracts were then centrifuged at 10,000*g* for 30 min at 4°C and the supernatant, containing the extracted proteins, was transferred into a fresh tube. Expression of FLAG-PCNA was confirmed with immunoblotting as previously described (Arroyo-Mateos et al., 2018). For mass spectrometry-based identification of the proteins that co-purify with Rep-GFP, 1 ml of total protein extract from each sample was incubated for 1 h with 25 μl (50% slurry) of GFP-Trap_M® beads (Chromotek) at 4°C. After incubation, the beads were captured with a magnetic rack and washed three times in 0.5 ml of washing buffer (10 mM Tris–HCl pH 7.5, 150 mM NaCl, 0.5 mM EDTA, 1 mM PMSF protease inhibitor). Antibodies used were anti-Flag (F7425; Sigma-Aldrich) and anti-GFP (3H9; Chromotek).

### Tryptic Digestion of the Immunopurified Proteins

The affinity purified proteins were subjected to on-bead tryptic digestion. Briefly, the GFP-Trap_M beads were washed twice with 400 μl of 50 mM ammonium bicarbonate buffer pH 8 (ABC), after which the beads were resuspended in 10 μl 50 mM dithiothreitol (DTT) in 50 mM ABC and incubated at 60°C for 1 h. Subsequentely, 12 μl of 50 mM iodoacetamide in 50 mM ABC was added and the sample incubated at room temperature in the dark for 1 h. Then, 14 μl 50 mM cysteine in ABC was added and the bound proteins were digested overnight at 20°C after adding 1 μl of a Trypsin (Roche, sequencing grade) solution (0.5 μg/μl in 1 mM HCl). Peptide digestion was terminated by acidification to pH3 by adding 1 μl 10% trifluoroacetic acid. The tryptic peptides were concentrated and cleaned using home-made μColumns (Lu et al., 2011). These μColumns were prepared by stacking in a 200 μl tip two 3M™ Empore™ C18 extraction disks and 4 μl of a LiChroprep^®^ RP-18 50% (Merck Millipor) slurry in methanol. The μColumns were washed with 100% methanol and equilibrated with 100 μl of 0.1% formic acid in water. Protein samples were added and eluted through the μColumns. The μColumns were washed with 0.1% formic acid in water and the bound peptides were eluted by adding 1:1 mixture of acetonitrile and 0.1% formic acid in water. The acetonitrile content of the samples was reduced by putting the samples in a centrifuge concentrator (Speed-Vac) at 45°C for 2 h and redissolved into 50 μl 1 ml/l formic acid in water.

### Mass Spectrometry Analysis and Data Processing

Digested peptides were analyzed using a nano LC-MS/MS LTQ-Orbitrap XL (Thermo Fisher), as previously described (Gawehns et al., 2015). All tryptic digests were sequentially run as one batch of samples on the nLC-MS machine to minimize technical variation. The MaxQuant software 1.5.2.8 (Cox and Mann, 2008; Hubner et al., 2010) was used to analyze the raw data from the LTQ-Orbitrap (Thermo Fisher) for protein identification and label-free quantification (LFQ). The Uniprot proteome database of tomato (UP000004994) and an *in-house* made database of contaminants (Peng et al., 2012) were included in the Andromeda search engine (Cox et al., 2014). The mass spectrometry proteomics data have been deposited to the ProteomeXchange Consortium *via* the PRIDE (Vizcaino et al., 2016) partner repository with the dataset identifier PXD018011.

Data filtering from the MaxQuant output was carried out with Perseus 1.5.5.3 (http://www.perseus-framework.org). Only the LFQ values of the proteins that were identified with at least two tryptic peptides, of which one should be unique and one unmodified, were log10 transformed for further analysis. Proteins that showed a ΔLFQ (log10 LFQ in the sample – log10 LFQ in the control) equal or higher than the ΔLFQ of FLAG-PCNA (*internal control*) in at least one out of three biological replicates were annotated as ‘putative interactors’ of Rep and included for further analysis.

### Computational Analysis

Gene ontology (GO) terms for biological process and cellular component were assigned to the co-purifying tomato proteins using Panther (Mi et al., 2017) and QuickGO (EMBL-EBI, https://www.ebi.ac.uk/QuickGO) tools. GO term enrichment was represented for every sample in a bar graph using Prism 7.0v (GraphPad) software. Venn diagrams were drawn using InteractiVenn (http://www.interactivenn.net/) (Heberle et al., 2015). A manually curated protein–protein interaction network was constructed with Cytoscape (Shannon et al., 2003). For that, the interaction network of every putative host interactor and the known Rep-interactors, as reported by Ruhel and Chakraborty (2019) were retrieved from the STRING protein database (https://string-db.org/). The resulting nodes (proteins) and edges (interactions) were arranged according to the force-directed layout. The protein sequence of the Arabidopsis gene model At4g28990 (closest Arabidopsis homologue of GRIEP1) was analyzed using the InterPro database (https://www.ebi.ac.uk/interpro/) to identify known protein domains in GRIEP1.

### Construction of Clones Used for Confocal Microscopy

All molecular DNA cloning techniques were performed using standard methods using (Sambrook et al., 2001). The *E. coli* strain DH5α was used for subcloning. The CDS of *EF1A* (XM_004251106; Solyc11g069700.1.1), *Nucleolin-like 2* (NM_001319854.1; Solyc02g014310.2.1), *Rep-interacting protein 1* (XM_004239224; Solyc05g018340.2.1) and *THO complex subunit 4A* (a.k.a. *SlALY1*) (Bruhn et al., 1997) (NM_001347950.1; Solyc10g086400.1.1) were amplified from tomato cDNA by PCR using Phusion DNA polymerase (Thermo Fisher) and the primers listed in **Table S1** that contained *attB1* and *attB2* recombination sites for Gateway-directed cloning. The resulting PCR products were recombined with the Gateway vector pDONR207 (Thermo Fisher) using BP Clonase II (Thermo Fisher) and the resulting pENTR plasmids confirmed by DNA sequencing. The cDNA clones were then introduced into the destination vectors pGWB452 (Nakamura et al., 2010) and pDEST-SCYNE^GW^ (Gehl et al., 2009) using LR Clonase II (Thermo Fisher). All plasmids generated and used in this work are listed in **Table S2**.

### Transient Expression in *N. benthamiana* by Agroinfiltration and Confocal Microscopy

For transient expression in *N. benthamiana* plants, the binary constructs were introduced in *Agrobacterium tumefaciens* GV3101, as previously described (Maio et al., 2019). Briefly, single colonies of *A. tumefaciens* were grown overnight to an OD_600_ of 0.8–1.5 in low salt (2.5 g/l NaCl) LB medium and resuspended in infiltration medium (1× MS [Murashige and Skoog] salts (Duchefa), 10 mM MES pH 5.6, 2% w/v sucrose, 200 μM acetosyringone) and incubated at room temperature for at least 2 h. Fully expanded leaves of 4-week old *N. benthamiana* plants were then syringe-infiltrated with these *A. tumefaciens* cell suspensions at an OD_600_ = 1 for all constructs. When two cultures were co-infiltrated for BiFC analysis, they were mixed at a ratio 1:1 to a final OD_600_ = 1. A*. tumefaciens* strain carrying the pBIN61 binary vector to express the P19 silencing suppressor (referred to as pBIN61:P19) of Tomato bushy stunt virus (TBSV) was added to every sample at a final OD_600_ = 0.5. Three days post-infiltration *N. benthamiana* leaf material was collected for microscopy. Subcellular localization of the GFP-tagged proteins and the reconstituted SCFP fluorophores was detected using a confocal laser scanning microscopy (Zeiss LSM510) with a c-Apochromat 40× 1.2 water-immersion Korr objective. The following beam/filter settings were used: GFP-excitation at 488 nm (argon laser), primary beam-splitting mirrors 405/488, secondary beam splitter 490 nm, band filter BP 505–550 nm; SCFP-excitation at 458 nm (argon laser), primary beam-splitting mirrors 458/514, secondary beam splitter 515 nm, band filter BP 470–500 nm. The experiments were repeated three times with a similar result.

### Virus-Induced Gene Silencing in *N. benthamiana*

For virus-induced gene silencing (VIGS), PCR-amplicons of 300 nucleotides length were designed (https://vigs.solgenomics.net/) (Fernandez-Pozo et al., 2015) and amplified from *N. benthamiana* cDNA for the closest homologues of the tomato candidate genes *GRIEP1* (Nbv6.1trP35301) and *SlALY1* (Nbv6.1trP36214) using the primers given in **Table S2**. These fragments were cloned into the *SmaI* site of pYL156 (Liu et al., 2002). The resulting vectors TRV2::*NbALY1* and TRV2::*NbGRIEP1* were confirmed by sequencing and transformed to *A. tumefaciens* GV3101. The different VIGS constructs were mixed in a 1:1 ratio with *A. tumefaciens* strain containing pTRV1 at a final OD_600_ of 0.8 per construct, as described (Liu et al., 2002). This bacterial suspension was syringe-infiltrated in the two first true leaves of two-week old transgenic *N. benthamiana* plants that contain the *2IR-35S:GFP* viral reporter cassette for monitoring TYLCV (isolate Almeria; AJ489258) replication and systemic movement (Maio et al., 2019). As negative control, a TRV2 construct was used that targets the non-plant gene *E. coli* β-glucuronidase (TRV2::*GUS*) (Tameling and Baulcombe, 2007), as the latter clone is less aggressive than TRV2 without insert. The VIGS experiment was repeated twice with 14 plants per silencing construct.

### Measurement of Viral Replication Activity of Rep in 2IR-GFP Plants

Two weeks post agroinoculation, one half of a fully expended systemic leaf was infiltrated with REP^TYLCV^-RFP, while the other half was infiltrated with KtoA triple mutant of REP^TYLCV^-RFP (K65A K69AK99A) at an OD_600_ of 1.0 (Maio et al., 2019). This latter KtoA triple mutant can no longer replicate the 2IR-GFP cassette (negative control). The replication efficiency of Rep of the viral 2IR-GFP cassette was then estimated indirectly using a radiometric method in which the ratio of RFP fluorescence (Rep levels) over the GFP fluorescence (GFP protein expression from extrachromosomal replisomes) was determined. To this end, in total eight leaf discs (each from a different plant) with a diameter of 5 mm were taken and placed in a 96-wells plate (Perkin-Elmer Optiplate™ 96) containing 100 µl double-distilled water three days post *A. tumefaciens* infiltration. Fluorescence was measured using microplate reader (BioTek Synergy™ H1 Hybrid multi-mode). GFP fluorescence was excited at 485 nm and the emission was measured at 528 nm (BP16 nm monochromator), while RFP fluorescence was excited at 555 nm and the emission measured at 583 nm (BP 16 nm monochromator). The data was compared by using a student t-test. The experiment was repeated twice with eight plants per silencing construct.

### Gene Expression Analysis Using Real Time PCR

In order to quantify the transcript levels of *NbALY1* or *NbGRIEP1*, a total of 100 mg of systemic leaf tissue was collected from 4-week old *N. benthamiana* plants (2-week post TRV inoculation). Following tissue grinding (Qiagen Tissuelyser II bead mill), total RNA was extracted using TRIzol LS (Thermo Fisher). RNA concentrations were determined measuring the absorbance at 260 nm (A260) using a NanoDrop (Thermo Fisher). A total of 1 µg of RNA was used for cDNA synthesis using RevertAid H reverse transcriptase (Thermo Fisher) following the manufacturer’s instructions. Real time PCR was performed with a QuantStudio™ 3 system (Thermo Fisher) using the EvaGreen kit (Biotium) according to the supplier’s instructions. Melting curves were analyzed to ensure amplification specificity and absence of primer-dimer formation. Primer PCR efficiencies were determined using a serial dilution of a mixed cDNA sample (1:2, 1:4, 1:8 & 1:16) and the qPCR reactions were conducted in duplicate (technical replicate) for four samples (biological replicates). Water and no-template controls were used as negative controls for each primer set. Cycle threshold (Ct) values were calculated using the auto baseline function (Quant studio software). Duplicates for which the Ct value different more than 0.5 were not considered and removed from the analysis. Finally, relative gene expression levels were calculated using the obtained Ct values using qBase+ (Biogazelle, Belgium) applying a method that corrects for primer efficiencies. Finally, the gene expression data was normalized using the *N. benthamiana* *APR* as reference gene (Liu et al., 2012). The VIGS experiment was repeated three times with a similar result. The *2IR-GFP* extrachromosomical molecules (ECMs) were quantified as described previously (Maio et al., 2019). Primers for the ECMs and the internal reference gene, *25S rRNA*, are given in **Table S2**. The qPCR and data analyses were performed using qBase+ software as described above.

### *N. benthamiana* Protein Extraction and Immunoblotting

Two leaf disks (approximately 50 mg) of *N. benthamiana* leaf material were harvested, snap frozen in liquid nitrogen and homogenized with plastic pestles. Laemmli buffer (0.1 M Tris pH 6.8, 20% glycerol, 4% SDS, 100 mM DTT, 0.001% Bromophenol blue) was added to each sample (100 μl of buffer per sample). Tubes were vortexed vigorously and heated for 10 min at 96°C. The extracts were then centrifugated at 14,000 rpm at 4°C for 5 min. A total of 10 μl of the protein extract was separated on 10% SDS-PAGE gels and subsequently transferred onto a PVDF membrane. Immunodetection of the proteins was performed according to standard protocols using anti-RFP antibody (Chromotek 6G6; 1:1,000) to detect the Rep-RFP fusion proteins, anti-GFP (Chromotek 3H9; 1:1,000) antibody for GFP and GFP-fusions as primary antibodies and anti-Rat (Pierce 31470; 1:10,000) or anti-Mouse (Pierce 31430; 1:10,000) as secondary antibodies. The labeled proteins were visualized using enhanced chemiluminescence (ECL, 0.1 M Tris–HCl pH 8.5, 1.25 mM luminol [Sigma-Aldrich 09253] in DMSO, 0.2 mM p-Coumaric acid [Sigma C9008] in DMSO, 0.01% H_2_O_2_) and detected using MXBE Kodak films (Carestream). Equal loading of the extracted proteins was confirmed by estimating the total amount of Rubisco in each sample using Ponceau S or Coomassie Brilliant Blue staining of the membrane.

## Results

### Identification of Novel Tomato Proteins That Interact With TYLCV Rep

**Figure 1:**
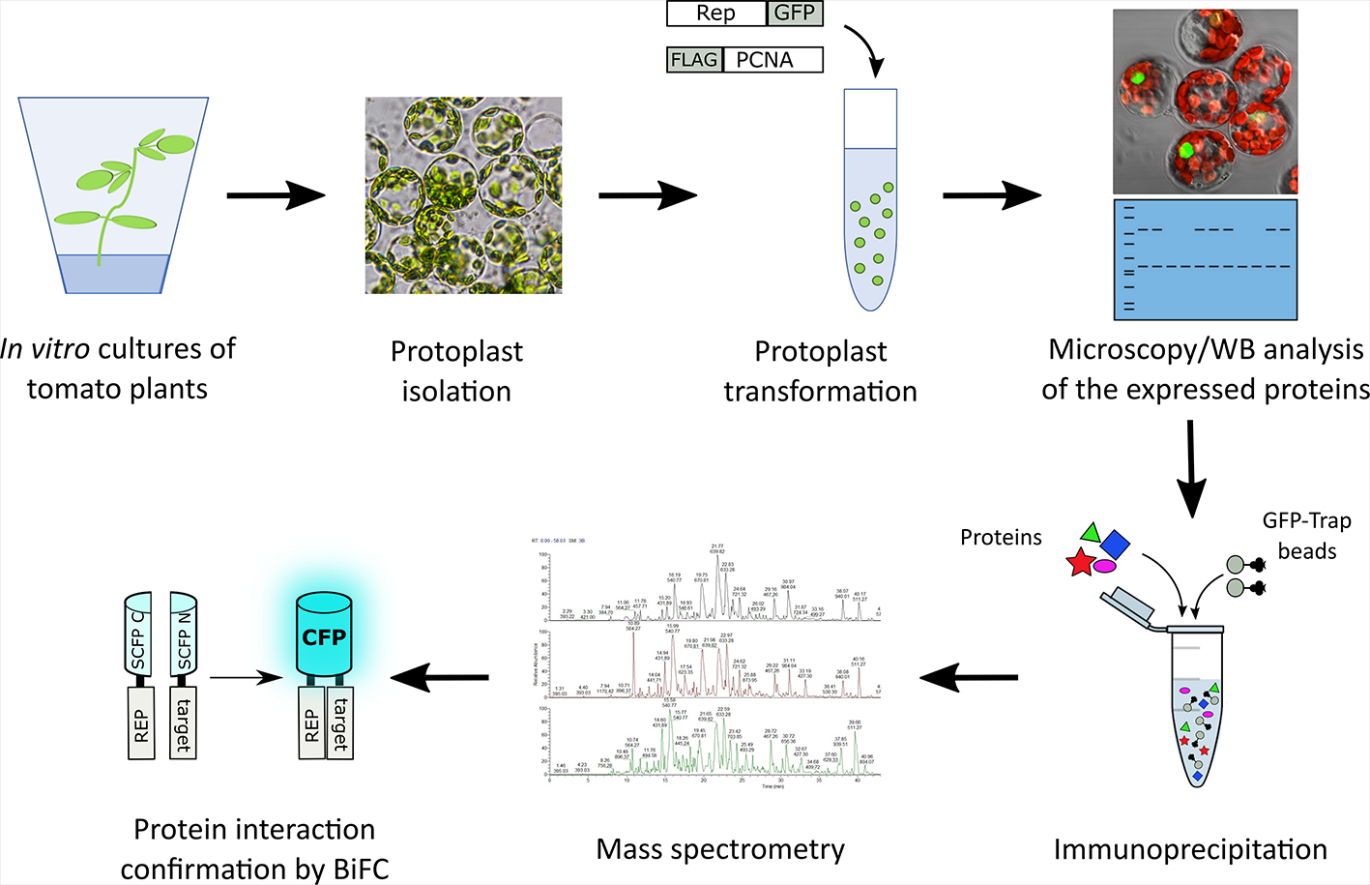
Schematic representation of the strategy followed to identify novel interactors of TYLCV Rep using affinity purification followed by mass spectrometry. Rep fused to GFP was expressed in the presence and absence of FLAG tagged PCNA in tomato protoplasts. The next day, the transfected protoplasts were inspected for a GFP signal using fluorescence microscopy and the total protein fraction was extracted, analyzed for the presence of the overexpressed proteins using immunoblotting (WB) and then subjected to affinity purification using anti-GFP resin (in three independent experiments). Co-purifying proteins were identified after tryptic digestion by tandem mass spectrometry (MS/MS). For a subset of interactors their interaction with Rep was confirmed using bimolecular fluorescence complementation (BiFC). The tryptic digests were prepared in parallel and the resulting peptide samples were analyzed as one batch of runs on the LC-MS.

**Figure 2:**
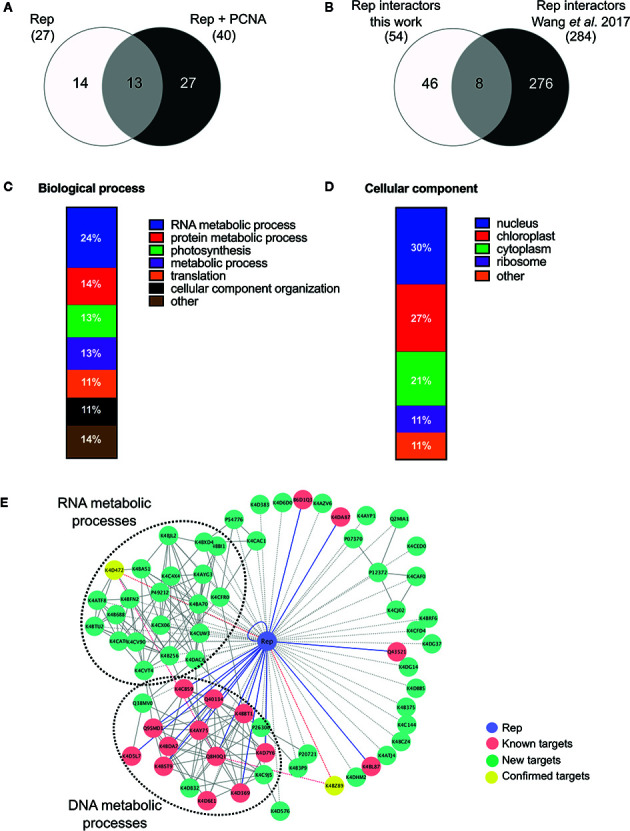
Functional annotation of the tomato interactomes of ‘Rep’ and ‘Rep+PCNA’. **(A)** Venn diagram depicting the number of interacting proteins that were enriched in the samples expressing ‘Rep’ alone and/or ‘Rep + PCNA’. **(B)** Venn diagram representing the number of Rep interactors that overlapped with a similar approach taken by Wang et al. (2017a), who purified TYLCV Rep from *N. benthamiana* cell extracts. **(C)** Bar chart depicting the proportion of proteins (%) in a subclass of the GO term “Biological process” for the tomato interactors of Rep, combining the two lists of interactors. **(D)** Bar chart showing the proportion of proteins (%) in a subclass of the GO term “Cellular component”, combining the two lists of interactors. **(E)** Protein network of the Rep tomato interactome identified in this work (green nodes) and retrieved from the literature (pink nodes). Yellow nodes represent targets whose interaction with Rep was confirmed in this work. Blue lines, known interactions with host proteins; dotted lines, putative interactions with host proteins; red lines, novel confirmed interactions; grey solid lines, protein-protein interactions between plant proteins.

In order to identify novel host proteins that interact with TYLCV Rep, we isolated tomato protoplasts from *in vitro* cultured plants and transfected them with plasmid DNA to express Rep-GFP alone or in combination with FLAG-PCNA (**Figure 1**). PCNA was co-expressed together with Rep to have an internal positive control for a Rep interacting protein, but also to promote assembly of viral replisomes. Accumulation of Rep-GFP was visually inspected in every sample by using fluorescence microscopy prior to the affinity purification, while FLAG-PCNA accumulation was confirmed by immunoblotting (**Figure S1**). The total protein fraction was extracted from three experimental replicates and subjected to affinity purification using anti-GFP resin followed by tryptic digestion in combination with a nano LC–MS/MS analysis. Identification and quantification of the (co)purifying proteins was then performed using MaxQuant. Rep was readily detected in every sample expressing Rep, while PCNA was enriched in the Rep-PCNA samples in comparison to the negative control (PCNA alone) in two out of three biological replicates. In total we identified 427 candidate interactors. To obtain a set of high-confidence interacting proteins, we used as selection criterion that their abundance after the pull-down should be similar or higher than PCNA in one of the replicates. This yielded a total of 54 tomato proteins (**Table 1**): 27 proteins in the sample expressing Rep-GFP alone, 40 in the sample in which Rep-GFP was expressed together with FLAG-PCNA, and 13 proteins were in present in both samples (**Figure 2A**). For eight of these interacting proteins, a close homolog was previously identified in a related pull-down experiment for TYLCV Rep in *N. benthamiana* (Wang et al., 2017a) (**Figure 2B** and **Table 2**).

**Table 1:** List of the putative interactors of Rep identified by affinity purifications/MS analysis. Proteins in bold were selected for further protein–protein interaction studies.

Protein ID(TrEMBL)	Functional gene description/annotation	Rep^1^	Rep-PCNA^2^
K4CJ02	Photosystem I reaction center subunit		+
K4DB32	Histone 2A	+	
Q2MIA1	P700 chlorophyll a		+
K4DG37	Glycosyltransferase	+	
**K4B6B8**	**Nucleolin-like**		+
K4ATJ4	Peptidylin-prolyl cis-trans isomerase	+	
K4D6D0	Putative Prohibitin-3		+
K4CVT4	MAR-binding protein		+
K4BCZ4	Protein CROWDED NUCLEI 4		+
K4BFN2	Small nuclear ribonucleoprotein Sm D3		+
**K4DAC6**	**Elongation factor 1-alpha**		+
P12372	Photosystem I reaction center subunit II		+
K4BPK3	Actin-41		+
K4B256	Glycine-rich RNA-binding protein RZ1A	+	
**K4D472**	**THO complex subunit 4A, *Sl*ALY1**		+
P26300	Enolase		+
K4C2T0	Chlorophyll a-b binding protein		+
K4CFD4	Aconitate hydratase		+
K4DHM2	Serine hydroxymethyltransferase	+	
K4CV90	Small nuclear ribonucleoprotein Sm D2		+
K4BXD4	Serine/threonine-protein phosphatase	+	+
K4B375	Prosequence protease 1		+
K4D383	NDR1/HIN1-like protein	+	
K4BTU2	Serine/arginine-rich splicing factor SC35	+	
K4AZV6	Rhodanese-like domain-containing protein 4		+
K4C9J5	Histone H2B	+	+
K4CAF0	Photosystem I reaction center subunit XI		+
K4BJL2	50S ribosomal protein L29	+	+
K4ATF8	Pre-mRNA cleavage factor Im		+
K4C144	Malic enzyme		+
K4BRF6	Putative methyltransferase	+	
P49212	60S ribosomal protein L37	+	+
K4BA51	H/ACA ribonucleoprotein complex subunit 4		+
**K4BZ89**	**RNA-binding protein EWS-like, GRIEP1**	+	
K4BBI1	50S ribosomal protein L12	+	
K4AYG3	50S ribosomal protein L1	+	+
P54776	26S proteasome regulatory subunit 6A		+
K4CAT6	Small nuclear ribonucleoprotein Sm D1		+
K4CUW3	60S ribosomal protein L23	+	+
K4C4X4	60S ribosomal protein L36	+	+
K4BA70	40S ribosomal protein S15		+
K4B3P9	Fructose-bisphosphate aldolase	+	
K4D576	Protein RNA-directed DNA methylation 3	+	+
K4AYP1	Dynamin-related protein 1E		+
Q2MIB8	30S ribosomal protein S16	+	+
K4CED0	Uncharacterized protein		+
K4DBB5	Zinc finger CCCH domain-containing protein	+	+
P07370	Chlorophyll a-b protein 1B		+
K4CX06	Ribosome biogenesis protein NSA2 homolog	+	
K4CAC1	26S proteasome regulatory subunit 6B	+	+
P20721	Low-temperature-induced cysteine proteinase	+	+
K4CFR0	26S proteasome non-ATPase regulatory subunit 2	+	
K4DG14	ATP synthase delta chain	+	
Q38MV0	Tubulin beta chain	+	+

^1^Found in the samples expressing REP-GFP alone.

^2^Found in the samples expressing REP-GFP together with PCNA.

**Table 2:** List of tomato interactors of TYLCV Rep here found that overlap with the study by Wang et al. (2017a).

*S. lycopersicum* ID (TrEMBL ID); this study	*N. benthamiana* gene model ID (Wang et al.)	Protein annotation
K4CJ02	NbS00018180g0004.1	Photosystem I reaction center subunit
K4BA70	NbS00016568g0015.1	40S ribosomal protein S15
K4BPK3	NbS00041237g0007.1	Actin-41
K4CFD4	NbS00008022g0007.1	Aconitate hydratase
K4CFR0	NbS00013228g0016.1	26S proteasome non-ATPase regulatory subunit 2
K4D6D0	NbS00032991g0022.1	Putative Prohibitin-3
K4DHM2	NbS00012020g0003.1	Serine hydroxymethyltransferase
P20721	NbS00040506g0007.1	Low-temperature-induced cysteine proteinase

### Functional Annotation and Protein Network Analysis of the Rep Interactome

In order to gain insight into the cellular processes affected by overexpression of TYLCV Rep in tomato protoplasts, a gene ontology (GO) analysis was conducted assigning functional categories to each interactor. The GO terms **“**Biological process**”** and **“**Cellular component**”** were assigned and the proportion of plant proteins per GO category (%) was calculated (**Table 3**). The largest GO category for **“**Biological process**”** was formed by proteins belonging to **“**RNA metabolism**”**, in particular the subclasses **“**involved in spliceosome and ribosome assembly**”**, **“**regulation of RNA polymerase**”** and **“**mRNA/rRNA processing**”** (**Figure 2C**). These categories were followed by **“**protein metabolic processes**”**, **“**photosynthesis**’**, **“**metabolic processes**”**, **“**translation**”** and **“**cellular component organization**”**. Only two GO categories were significantly overrepresented (*p*-value **<**0.05), i.e. **“**RNA metabolic processes**”** and **“**metabolic processes**”**. In total 30% of the identified proteins was assigned to be nucleus based on their annotation (**Figure 2D**). In order to visualize the Rep-interactome, we plotted the 54 tomato proteins in a protein interaction network (**Figure 2E**) with the nodes representing (i) the newly identified host proteins (green and yellow), (ii) 16 Rep-interactors derived from previous studies (pink) (reviewed by Ruhel and Chakraborty, 2019) and (iii) Rep (blue). In total, seventy known interactions with Rep (Ruhel and Chakraborty, 2019) were included in this protein network (blue edges and dotted edges) and a total of 148 plant protein interactions (grey edges) were retrieved from the STRING database (**Table S3**). This network contained two major clusters. The first cluster comprises (mostly) known Rep interactors involved in DNA metabolism, such as DNA replication and repair, while the second cluster is primarily formed by newly-identified candidates implicated in RNA metabolic processes, which hints to that Rep potentially regulates RNA biogenesis in an unknown manner.

**Table 3:** List of gene ontology classes assigned to the identified tomato proteins.

GO ID	Biological process	
	GO term	Protein identifiers (TrEMBL ID)
**GO:0016070**	RNA metabolic processes	K4CVT4, K4CAT6, K4BFN2, K4CV90, K4BZ89, K4D576, K4ATF8, K4CX06, K4AYG3, K4BA51, K4BTU2, K4D472, K4DBB5
**GO:0019538**	protein metabolic processes	K4B375, K4CFR0, P54776, K4CAC1, P20721, K4BXD4, K4DAC6
**GO:0015979**	photosynthesis	K4CJ02, K4CAF0, P12372, Q2MIA1, A0A0J9YZP9, P07370, K4DG14
**GO:0008152**	metabolic processes	K4DG37, K4CFD4, K4C144, K4B3P9, P26300, K4DHM2, K4AZV6
**GO:0006412**	translation	P49212, K4BJL2, Q2MIB8, K4CUW3, K4BBI1, K4C4X4
**GO:0016043**	cellular component organization	K4BPK3, K4BCZ4, K4BA70, K4B6B8, K4AYP1, Q38MV0
**GO:0006259**	DNA metabolic process	K4CED0, K4DB32, K4C9J5
**_**	Other	K4ATJ4, K4BRF6, K4D383, K4D6D0, K4B256
**GO ID**	**Cellular component**		
	**GO term**	**Protein identifiers (TrEMBL ID)**
**GO:0005840**	ribosome	K4BJL2, K4BBI1, K4BA70, K4CUW3, P49212, K4C4X4,
**GO:0005634**	nucleus	K4CED0, K4CFR0, K4C9J5, K4ATF8, K4D576, K4CAT6, K4BFN2, K4CV90, K4BZ89, K4BTU2, K4B6B8, K4B256, K4BXD4, K4BCZ4, K4CX06, K4CVT4, K4DB32
**GO:0005737**	cytoplasm	K4AYP1, K4BRF6, K4DAC6, Q38MV0, P26300, K4BA51, K4CFD4, P20721, K4DHM2, K4B6B8, K4B256, K4BXD4
**GO:0009507**	chloroplast	K4C144, Q2MIB8, K4AYG3, P07370, K4DG14, A0A0J9YZP9, Q2MIA1, K4CJ02, P12372, K4B375, K4B3P9, K4ATJ4, K4D472, K4BCZ4, P20721
**_**	Other	K4DBB5, K4AZV6, K4D383, K4D6D0, K4CAF0,K4DHM2

### Examples of Novel Rep Interactors Linked to Viral DNA Replication and RNA Metabolic Processes

Our list of TYLCV Rep interactors contained several proteins linked to DNA replication and pathogenesis. For example, Elongation factor 1-alpha (EF1A), found in the sample Rep-PCNA, participates in cellular functions like translation, nuclear export, transcription but also apoptosis in virus-infected cells (reviewed in Sasikumar et al., 2012). EF1A is also involved in the propagation of certain positive-strand RNA viruses, as it has been found to interact with viral RNA and certain virus-encoded RNA-dependent RNA polymerases (Abbas et al., 2015).

Another putative novel Rep interactor detected in the AP-MS with Rep-PCNA is Nucleolin-like 2, a major nucleolar protein that regulates the rDNA chromatin structure, rRNA gene expression, pre-rRNA processing and assembly of ribosome particles (Durut and Sáez-Vásquez, 2015). Interestingly, Nucleolin interacts also with PCNA and the Replication protein A 32 (RPA32) during replication stress in human cell lines (Kawamura et al., 2019). These authors also provided evidence that Nucleolin is recruited to damaged replication forks and that recruitment of Nucleolin controls the activation of homologous recombination, a process that is also promoted by geminiviruses in infected cells (Richter et al., 2014). These observations argue for a functional link between Rep and Nucleolin-like 2. Possibly related, the coat protein (CP) of TYLCV was shown to be recruited to the nucleolus in the absence of the virus, while in the presence of the virus it was recruited to discrete nuclear foci (Rojas et al., 2001; Wang et al., 2017b). Expression of Rep was already sufficient to exclude CP from the nucleolus, but its localization in foci was only seen during a TYLCV infection (Wang et al., 2017b). In fact, a plethora of animal and plant viruses but also virus-encoded proteins reside in the nucleolus where they interact with nucleolar proteins and several viral proteins have been shown to co-localize with, reorganize and re-distribute nucleolar proteins such as Nucleolin (Hiscox, 2002; Kim et al., 2004; Chaudhry et al., 2018).

A third interactor again coming from the list of proteins co-purifying with Rep-PCNA and implicated in host-virus interactions is the THO subunit 4A, which is part of the THO core complex of the TREX (TRanscription-EXport) complex. This complex is conserved across eukaryotes and is linked to nuclear export of mRNA (Heath et al., 2016; Sørensen et al., 2017). The THO/TREX complex is targeted by various viral proteins to redirect and control viral mRNA translocation (Schneider and Wolff, 2009; Yarbrough et al., 2014). The closest Arabidopsis homologue of the tomato interactor here identified (THO subunit 4A) is the mRNA export adaptor protein ALY1 (AT5G59950). Once bound to mRNA, the ALY proteins interact with the nuclear export receptor, which then facilitates export of the bound mRNA. Interestingly, compromised *ALY1* gene function in Arabidopsis results in loss of RNA-dependent DNA methylation (RdDM) due to deficient export of *Argonaute6* mRNA (Choudury et al., 2019). In line with this connection, Rodríguez‐Negrete and co-workers (2013) demonstrated that TYLCSV Rep displays transcriptional gene silencing (TGS) suppressor activity by interfering with the host DNA methylation machinery during infection. In particular, Rep down regulates the transcript levels of key enzymes that maintain this modification, namely the DNA methyltransferases *MET1* and *CMT3*. Arguably, TYLCV Rep might thus inhibit tomato ALY1 function to modulate RdDM activity.

Finally, our list of candidate interactors that interacted with Rep in the absence of PCNA overexpression contained an uncharacterized protein, here called Geminivirus Rep interacting EWS-like protein 1 (GRIEP1), that shows sequence homology to the human RNA-binding protein EWS (Ewing Sarcoma protein). This mammalian protein acts as transcriptional repressor that regulates pri-miRNA processing and plays a role in tumorigenic processes (Ohno et al., 1994; Ouyang et al., 2017). The closest homolog of GRIEP1 in Arabidopsis is At4g28990, a predicted 395 amino acid protein with a predicted Zinc finger domain of the RanBP2-type that groups with the multifunctional TET protein family (TAF15/EWS/TLS; Interpro IPR034870) (Law et al., 2006). This protein family is implicated in transcription, (alternative) splicing, mRNA transport but also DNA repair (Kovar, 2011). To our knowledge this protein has not been studied in any plant species yet. Based on their potential role on DNA replication and/or Rep activity, we selected these four proteins to corroborate their interaction *in planta* (bold in **Table 1**).

### Confirmation of the Interaction Between Rep and the Putative Interactors

**Figure 3:**
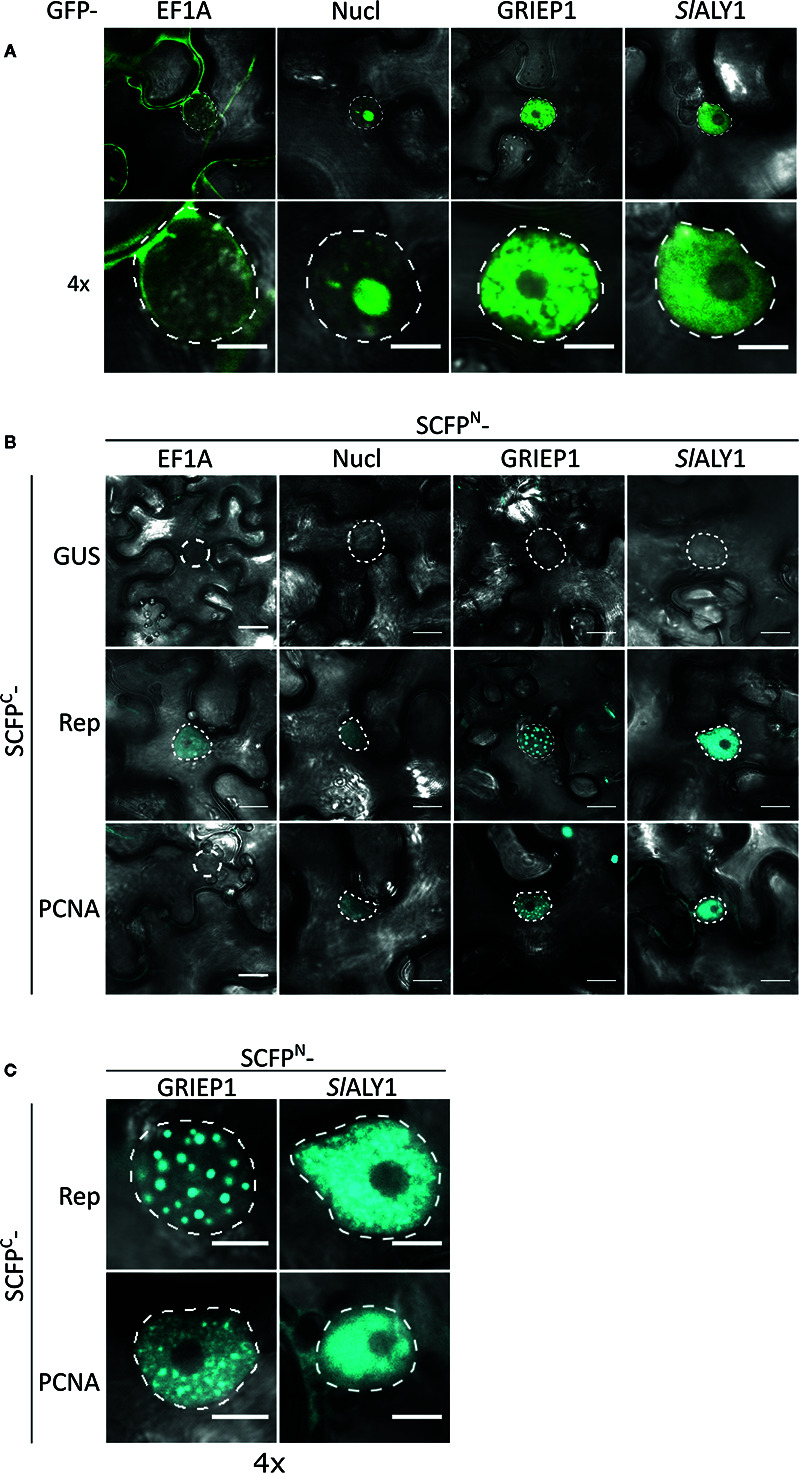
Cellular localization and BiFC protein interaction assay of the Rep-interacting proteins EF1A, Nucleolin-like 2, GRIEP1 and THO. **(A)** Subcellular localization of GFP-tagged tomato EF1A, Nucleolin-like 2 (Nucl), GRIEP1 and *Sl*ALY1 proteins in *N. benthamiana* epidermal cells upon transient overexpression. Image shown represents a typical cell (top) and a 4× zoom showing only its nucleus (bottom). **(B)** In a BiFC assay, EF1A and Nucleolin-like 2 do not seem to interact with TYLCV Rep nor PCNA since only a faint background fluorescence signal was detected, whereas GRIEP1 and *Sl*ALY1 show strong reconstitution of SCFP fluorophore in combination with Rep and PCNA in nuclei of the transfected cells. **(C)** 4× zoom of the nuclei of the positive BiFC interaction pairs shown in **(B)**. Scale bar is 5 μm for **(A, C)** and 20 μm for **(B)**. Dotted lines outline the nucleus. These experiments were repeated three times independently; for each combination, a minimum of 50 cells was analyzed in each experiment.

To assess in an independent assay the specificity of the interactions, we used bimolecular fluorescence complementation (BiFC), as it also provides information on where the interaction occurs in the cell. First, we assessed their subcellular localization pattern in *N. benthamiana* expressing GFP-labelled variants of these proteins. As expected, GFP-EF1A localized to the cytoplasm, while GFP-Nucleolin-like 2 localized exclusively to the nucleolus (**Figure 3A**). *Sl*ALY1 was uniformly distributed in the nucleoplasm with occasionally some small nuclear speckles. GRIEP1 resided, however, in large nuclear bodies (or aggregates) of unknown function or origin. Using immunoblotting, we confirmed that these GFP-fusions accumulated at the expected protein mass in *N. benthamiana* (**Figure S2**). We then fused the N-terminal half of the super Cyan fluorescent protein (SCFP^N^) to the N-terminus of the candidate proteins and the resulting fusion proteins were expressed in *N. benthamiana* epidermal cells together with Rep or PCNA fused to the C-terminal half of SCFP (SCFP^C^) (**Figure 3B**). As negative control, the SCFP^N^ fusion proteins were co-expressed with a non-plant protein (SCFP^C^-tagged β-Glucuronidase from *E. coli*). We detected no BiFC signal for any of these four interactors in combination with GUS in any cellular compartment. For SCFP^N^-EF1A and SCFP^N^-Nucleolin-like 2, only a faint SCFP signal was observed in some nuclei in combination with TYLCV Rep or PCNA. In contrast, the BiFC couple GRIEP1-Rep showed a strong and specific CFP fluorescence signal in distinct (large) nuclear bodies (NBs) and, to a minor extent, in the cytoplasm. Also, the combination of GRIEP1 and PCNA yielded a strong CFP fluorescence signal in the nucleus with some speckles and a weaker fluorescence signal in the cytoplasm (**Figures 3B, C**). Finally, *Sl*ALY1 interacted with Rep in the nucleoplasm and with PCNA in both the cytoplasm and nucleoplasm. These BiFC experiments thus corroborate our MS data that GRIEP1 and *Sl*ALY1 interact specifically and directly with Rep.

Next, we used VIGS to assess whether reduced transcript levels of these two confirmed Rep-interactors (GRIEP1 and *Sl*ALY) would alter DNA replication activity of Rep. To this end, we took advantage of *N. benthamiana* expressing a *2IR-GFP* reporter cassette (Maio et al., 2019), which allows monitoring of viral replication *in situ* as a result of TYLCV Rep expressio,. The VIGS constructs caused indeed effective gene silencing of *SlALY1* and *GRIEP1* without negatively impacting the plant development (**Figures S3A, B**). Two weeks post TRV inoculation, Rep-RFP was transiently expressed in a fully expanded leaf and 3 days later replication of the viral reporter was (indirectly) quantified by comparing the signal intensities of the GFP and RFP fluorescence (**Figure S3C**). Plants silenced for *GRIEP1* or *SlALY1* did not exhibit any reduced Rep replication activity, as measured either by accumulation extrachromosomal circular DNA molecules (ECMs) that derive from the *2IR-GFP* cassette or GFP fluorescence (**Figures S3E, F**). In fact, silencing of *GRIEP1* did significantly increase ECM accumulation compared to GUS silenced plants (*p*-value = 0.0137), and silencing of *ALY1* resulted in significantly increased GFP intensity (nearly 2.5-fold increase). These data thus argue that both *GRIEP1* and *ALY1* might act as inhibitors of Rep DNA replication activity, but we cannot exclude that they impact functions of Rep other than replication activity. Future work should reveal if overexpression of GRIEP1 and ALY1 can repress Rep activity.

## Discussion

This work describes an AP-MS proteomics approach to identify novel host proteins that (in)directly interact with TYLCV Rep and to explore their role for geminivirus replication. This yielded 54 putative interactors of TYLCV Rep of which 40 were detected in cells overexpressing Rep together with tomato PCNA and two were confirmed to interact with both Rep and PCNA in the BiFC assay. The majority of the interactors are involved in RNA biogenesis, such as spliceosomal complex assembly, regulation of DNA-dependent RNA polymerase, rRNA expression and maturation and RNA binding, or in protein metabolic processes like catabolic activity, enzyme regulation and proteasome complex. These findings align in part with the proteomics results obtained by Wang and co-workers (2017a), who found that their list of TYLCV Rep interactors in *N. benthamiana* was enriched for the GO terms belonging to **‘**protein catabolic processes**’** but not for **‘**RNA metabolism**’**. Moreover, for eight of the Rep interactors here identified a close homolog was found to interact with TYLCV Rep in *N. benthamiana* (Wang et al., 2017a). Several arguments support the validity of our findings. First, we were able to specifically co-purify PCNA with Rep in two out of three of the independent affinity purifications compared to the negative control, demonstrating that the pulldown conditions were suitable to identify interactors of Rep. In addition, some of the AP-MS hits found were previously found to interact with Rep, e.g. the Histones 2A and 2B (Kong and Hanley-Bowdoin, 2002). Second, a substantial proportion of the putative Rep-interactors were predicted to be nuclear localized *in silico.* This agrees with the known subcellular localization of Rep and the viral replisome (Kushwaha et al., 2017; Maio et al., 2019). Third, we were able to confirm the interaction between Rep and two selected candidates *in planta* using the BiFC system and to detect formation of this complex in the nucleus. These two proteins also interacted with PCNA in our BiFC, suggesting that they might be part of viral replisomes or transiently interact with them. GRIEP1 is apparently not required for the Rep-mediated viral DNA replication activity, as the latter remained unchanged in *GRIEP1*-silenced plants. As well, it was not possible from our experiment to unambiguously determine whether Rep activity was altered as a result of *SlALY1*-silencing in *N. benthamiana*. Future studies should reveal if this latter interaction is really involved in viral replication or suppression of transcriptional gene silencing, as reported for other viral proteins (Xu et al., 2020).

Despite the identification of novel interactors of TYLCV Rep in tomato, several limitations apply to our approach. First, we also detected chloroplastic proteins implicated in photosynthesis and proteins involved in ribosome assembly and biogenesis. As a connection is lacking between Rep and photosynthesis and given the difference in compartmentalization of these processes, these proteins likely represent false positives. In fact, photosynthesis-related and ribosomal proteins are highly abundant plant proteins and as such common contaminants picked up in proteomics methods such as AP-MS (Smaczniak et al., 2012). At the same time, many true interactors could easily have been missed due to the stringency of our selection criterium, low protein levels in non-dividing mesophyll cells, weak or transient interactions with Rep or the presence of the tag on Rep, which all could prevent detection of protein-protein interactions. The identified host proteins may also indirectly bind to TYLCV Rep. For example, for Elongation factor 1A and Nucleolin-like 2, we were not able to confirm their interaction using BiFC. This notion of an indirect interaction is corroborated by the observation that, in our interaction network, EF1A is connected to two known interactors of Rep, Ubiquitin conjugation enzyme 2 (UBC2) and Histone 3 (H3). The last aspect to consider is that our experimental results derive from the analysis of epidermal tomato protoplasts that express Rep in the absence of a viral infection. To obtain a more comprehensive picture of the host proteins that interact with Rep, the interactions should also be studied in the context of a virus-infection in phloem cells.

Interestingly, two RNA-binding proteins were confirmed as interacting proteins of both Rep and PCNA. The first one, GRIEP1, is an unknown conserved protein with an EWS-like RNA-binding protein that contains a Zinc finger domain of the RanBP2 type. Interestingly, in Arabidopsis *GRIEP1* is mostly expressed in the shoot apex and floral tissues (data available on ePlant, http://bar.utoronto.ca/eplant/), where meristematic cells are dividing, suggesting that the Arabidopsis homolog has a potential role in cell division or differentiation. Moreover, the BiFC couple between Rep and this EWS-like protein resided in nuclear bodies (NBs). We reported previously that the BiFC pair Rep-SCE1 (SUMO E2 conjugating enzyme 1) also aggregates in NBs (Maio et al., 2019). Co-localization studies with marker proteins of different NBs should elucidate the nature and biological function of these different NBs.

The other confirmed interactor is a subunit of the THO complex, *Sl*ALY1. The biological function of this THO/TREX complex is well characterized in eukaryotes, where it is recruited to nascent mRNA, where it connects transcriptional elongation to export of mature mRNA (Jimeno et al., 2002; Strässer et al., 2002). This complex is also essential for the nuclear export of the Kaposi**’**s sarcoma-associated herpes virus mRNAs and for viral DNA replication (Boyne et al., 2008). In plants, the THO/TREX complex has been shown to influence the production of trans-acting small interfering RNAs (Yelina et al., 2010) and a component of this complex appears to be involved in plant disease resistance against powdery mildew pathogen (Pan et al., 2012). As Rep is known to act as suppressor of RNA silencing (Liu et al., 2014), additional studies are needed to elucidate the mechanism and biological consequence of this Rep-THO/TREX interaction.

## Data Availability Statement

The mass spectrometry proteomics data have been deposited to the ProteomeXchange Consortium *via* the PRIDE (Vizcaino et al., 2016) partner repository with the dataset identifier PXD018011.

## Author Contributions

FM, HB, and MP designed the experiments. FM performed the protoplast isolations and transfections, and prepared the protein samples for nLC-MS/MS measurements. SB performed the nLC-MS/MS measurements. FM and SB analyzed the MS/MS data. FM, TH, MW, and MA-M performed the cloning, agroinfiltration, microscopy analysis, silencing, and qPCR analysis. FM, HB, and MP wrote the manuscript. All authors contributed to the article and approved the submitted version.

## Funding

The Topsector T&U program Better Plants for Demands (grant 1409-036 to HB), including the partnering breeding companies, supported this work. FM was financially supported by Keygene N.V. (The Netherlands).

## Conflict of Interest

Author MP is employed by the company Keygene N.V.

The remaining authors declare that the research was conducted in the absence of any commercial or financial relationships that could be construed as a potential conflict of interest.
