# Supplementary material for: Identification of Tomato Proteins That Interact With Replication Initiator Protein (Rep) of the Geminivirus TYLCV
Source: Front Plant Sci. 2020 Jul 15;11:1069. doi: 10.3389/fpls.2020.01069 (PMC7373745; doi:10.3389/fpls.2020.01069)
Supplement: Supplementary file 1 [file Presentation_1.pdf]

Supplementary Material

1 Supplementary Figures

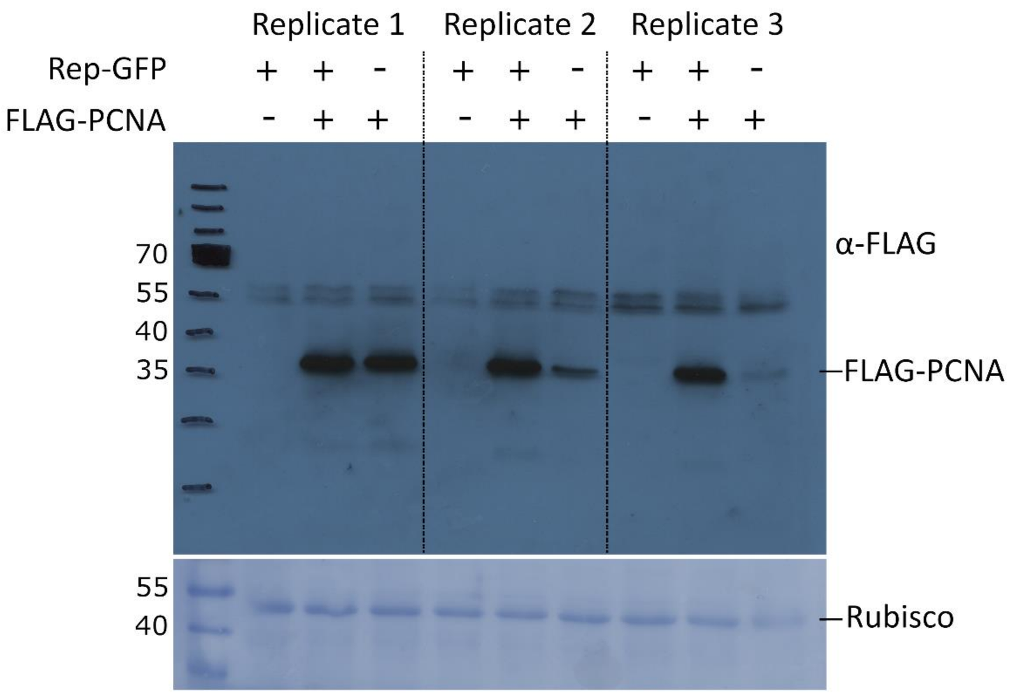

Supplementary Figure S1. immunoblot analysis of the PCNA protein levels upon transient expression in tomato protoplasts.

FLAG-tagged PCNA was detected with an anti-FLAG antibody in total protein extracts separated on SDS-PAGE followed by immunoblotting. Total protein fraction was extracted from three independent protoplast transfections. The GFP-based affinity purification and subsequent tryptic peptide digestion on the co-purifying proteins was executed in parallel on all protoplast samples generated. The resulting peptide digests were then analyzed as one batch run on the nLC-MS to minimize technical variation, as detailed in Figure 1. To confirm equal protein extraction and protein loading on the gel, membrane was stained with Coomassie Brilliant Blue (loading control).

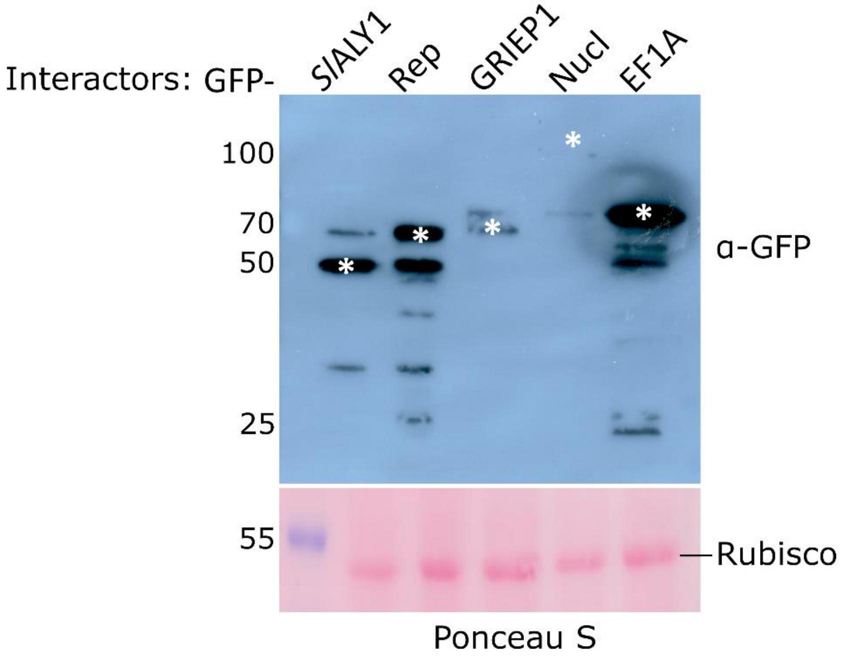

Supplementary Figure S2. Detection of the GFP-tagged interactors of TYLCV Rep upon transient expression in *N. benthamiana.*

To confirm expression of the GFP-fusion proteins (top) at the expected mass, the total protein fraction was extracted from leaves 3 days post *Agrobacterium* infiltration, separated on SDS-PAGE and transferred to a membrane. The GFP-tagged proteins were detected using chemiluminescence using an anti-GFP antibody followed by an HRP-conjugated goat anti-mouse secondary antibody. The asterisks mark the expected protein bands at the correct apparent mass for each protein sample. *Top:* construct infiltrated. *Left:* protein markers (kDa). To confirm equal protein loading, the membrane was stained with Ponceau S staining to reveal Rubisco.

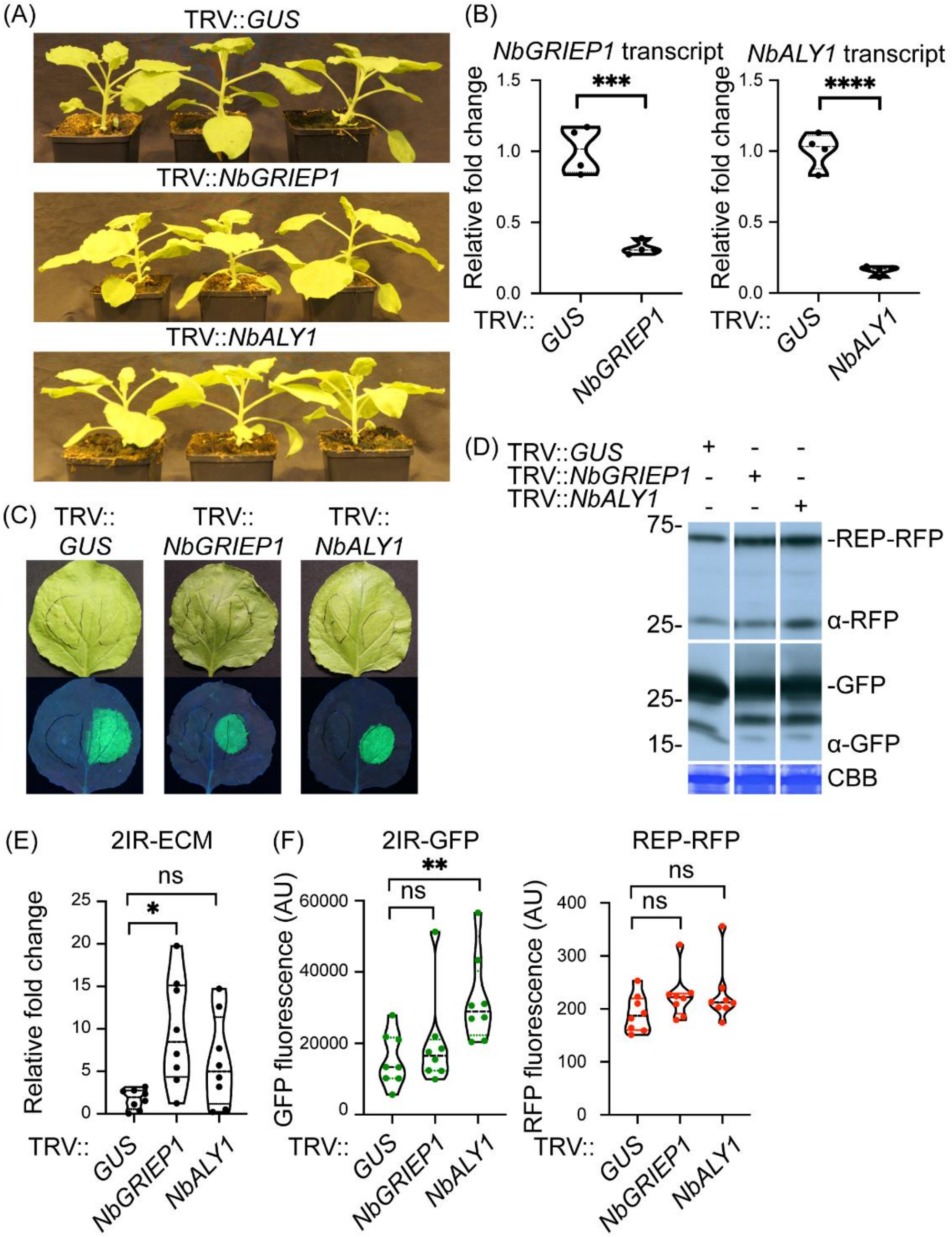

Supplementary Figure S3. Effect of gene silencing of *RIP1* and *SlALY1* on Rep viral DNA replication activity in *N. benthamiana* plants using a *2IRGFP* reporter cassette.

(A) Growth phenotype of 4-week-old *N. benthamiana 2IRGFP* plants inoculated with TRV to induce gene silencing of *GUS* (negative control, top), *NbGRIEP1* (middle), and *NbALY1* (bottom). Experiment was repeated three times with similar result (14 plants per construct).

(B) Transcript levels of the targeted genes *NbGRIEP1* and *NbALY1* in the TRV-inoculated plants: Left panel: TRV::*GUS*, TRV::*NbGRIEP1*; Right panel: TRV::*GUS*, TRV:: *NbALY1*. Samples (n=4 biological replicates) were taken from plants shown in panel A. Unpaired student's t-test (***, p-value <0.01; ****, p-value <0.001).

(C) Natural light (*top*) and UV image (*bottom*) of *N. benthamiana 2IRGFP* leaves revealing GFP accumulation due to extrachromosomal DNA replication of *2IRGFP* by Rep activity and subsequent expression of the extrachromosomal encoded *35S::GFP* gene. Four-week-old TRV-silenced plants (*GUS*, *NbGRIEP1*, *NbALY1*) were agroinfiltration to express Rep-RFP, wild type (right side) or the triple KtoA mutant (left side; inactive Rep variant). Images were taken of six individual leaves of six plants 3 days post agroinfiltration.

(D) Immunoblot of total protein extract from agroinfiltrated leaves (panel C) to determine Rep (anti-RFP) and GFP (anti-GFP) protein levels. To confirm equal protein loading, the membranes were stained with Coomassie Blue Brilliant (uncropped film is shown in Supplemental Figure S4).

(E) Quantification of circular extrachromosomal *2IRGFP* molecules (ECMs) by means of qPCR. The ECMs levels were normalized to an internal chromosomal gene fragment (DNA encoding the *25S rRNA* gene) (n=8 plants per TRV construct). The relative abundance of the ECMs were compared to GUS silenced control plants. Two-way ANOVA with a Dunnett's multiple comparisons test (*, p-value <0.05; ns, not significant).

(F) Quantification of the GFP and RFP fluorescence signals as a proxy for Rep DNA replication activity and Rep protein levels, respectively, in leaf discs of *N. benthamiana 2IR-GFP* for the plants shown in panel C (n=8 plants per TRV construct). Student's t-test (**, p-value <0. 01; ns, not significant). Experiment was repeated three times with similar result.

2 Supplementary Tables

Table S1. Primers used in this study. Primer numbers refer to our internal database.

Primer number	Primer name	DNA sequence (5' to 3')
**7522**	EF1A attB1 Fw	GGGGACAAGTTTGTACAAAAAAGCAGGCTTCATGGGTAAAGAGAAGGTTCA
**7523**	EF1A attB2 Rv	GGGGACCACTTTGTACAAGAAAGCTGGGTCTCATTTTTTCTTCTGAGCAG
**7567**	Nucl_064 attB1 Fw	GGGGACAAGTTTGTACAAAAAAGCAGGCTTCATGGGTAAATCTATCAAGAAG
**7568**	Nucl_064 attB2 Rv	GGGGACCACTTTGTACAAGAAAGCTGGGTCTTACTCGTCACTAAAGGTAGTC
**7614**	Fw_GRIEP1_X2_attB	GGGGACAAGTTTGTACAAAAAAGCAGGCTTCATGGGTTCGCGTGAGAAGG
**7615**	Rv_GRIEP1_X2_attB	GGGGACCACTTTGTACAAGAAAGCTGGGTCCTAATATGCACCTCGTCCA
**7692**	Fw_THO4A_attB	GGGGACAAGTTTGTACAAAAAAGCAGGCTTCATGTCAAATCTTGATGTATC
**7693**	Rv_THO4A_attB	GGGGACCACTTTGTACAAGAAAGCTGGGTCTTAGTTTGTCTGCATGGCTT
**8811**	VIGS NbALY1 FW	ACAGTACATGGGACCACAAC
**8812**	VIGS NbALY1 RV	GGCTTTCCATCTAGCTGAAC
**8813**	VIGS NbRIP1 Fw	GGATATAGAATACGTGCAGG
**8814**	VIGS NbRIP1 rv	ATCCATGCACATCCAATCAC
**9105**	qPCR NbALY1 FW	ACAGTACATGGGACCACAAC
**9106**	qPCR NbALY1 RV	GGCTTTCCATCTAGCTGAAC
**9107**	qPCR NbGRIEP1 Fw	GGATATAGAATACGTGCAGG
**9108**	qPCR NbGRIEP1 rv	ATCCATGCACATCCAATCAC
**8355**	qPCR NbAPR FW	CATCAGTGTCGTTGCAGGTATT
**8356**	qPCR NbAPR RV	GCAACTTCTTGGGTTTCCTCAT
**7981**	Nb25SrRNAFw	ATAACCGCATCAGGTCTCCA
**7982**	Nb25SrRNARv	CCGAAGTTACGGATCCATTT
**8031**	ECMs2Fw	CTGGGGACCTGCAGGC
**8032**	ECMs2Rv	CGCGCGGTGTCATCTATGTTAC

Table S2. Plasmids used in this study. The identifier refers to our internal database

Identifier	Gene fragment	Plasmid backbone	Protein tag	Described in or here generated
**bgIFP5568**	EF1A	pENTR207	-	PCR amplicon (primers 7522/7523)
**pFP1696**	Nucleolin	pENTR207	-	PCR amplicon (primers 7567/7568)
**bgIFP5569**	EWS, GRIEP1	pENTR207	-	PCR amplicon (primers 7614/7615)
**bgIFP5570**	THO4A	pENTR207	-	PCR amplicon (primers 7692/7693)
**pFP1194**	-	pGWB452	GFP-	*Nakamura et al. 2010*
**pFP1412**	-	pDEST-SCYNE(R)-gw	$SCFP^N$-	*Gehl et al. 2009*
**pFP1415**	-	pDEST-^{GW}SCYCE	-$SCFP^C$	*Gehl et al. 2009*
-	-	pK7FWG2	-EGFP	*Karimi et al. 2002*
**bgIFP5560**	TYLCV Alb13 Rep-GFP	pK7FWG2	-EGFP	Keygene N.V.
**bgIFP5571**	FLAG-PCNA	pJTRBO	FLAG-	*Arroyo-Mateos et al. 2018*
**bgIFP5368**	TYLCV Rep-$SCFP^C$	pDEST-^{GW}SCYCE	-$SCFP^C$	*Maio et al. 2019*
**bgIFP5401**	PCNA-$SCFP^C$	pDEST-^{GW}SCYCE	-$SCFP^C$	*Arroyo-Mateos et al. 2018*
**bgIFP3944**	$SCFP^C$-GUS	pDEST-SCYCE ^{GW}	$SCFP^C$-	PCR amplicon
**bgIFP6270**	-	pTRV1	-	*Liu et al. 2002*
**bgIFP6268**	TRV2::*GUS*	pYL156	-	*Tameling and Baulcombe 2007*
**BgIFP6264**	TRV2::*ALY1*	pYL156	-	PCR amplicon (primers 8811/8812)
**bgIFP6265**	TRV2::G*RIEP1*	pYL156	-	PCR amplicon (primers 8813/8814)
**bgIFP4981**	TYLCV REP-RFP	pGWB654	-mRFP	*Maio et al. 2019*
**bgIFP5420**	TYLCV REPKtoA-RFP	pGWB654	-mRFP	*Maio et al. 2019*
